# Transoral endoscopic head and neck surgery (eHNS) for minor salivary gland tumors of the oropharynx

**DOI:** 10.1186/s41199-017-0024-2

**Published:** 2017-05-31

**Authors:** David W. Schoppy, Michael E. Kupferman, Amy C. Hessel, Diana M. Bell, Elizabeth M. Garland, Edward J. Damrose, F. Christopher Holsinger

**Affiliations:** 1 Division of Head and Neck Surgery, Department of Otolaryngology, Stanford Cancer Center, Stanford University School of Medicine, 875 Blake Wilbur Drive, CC-2227, Palo Alto, CA 94304 USA; 2 Department of Head and Neck Surgery, The University of Texas M.D. Anderson Cancer Center, Houston, TX USA

**Keywords:** Transoral robotic surgery, Endoscopic head and neck surgery, Minor salivary gland tumors

## Abstract

**Background:**

Transoral endoscopic head and neck surgery (eHNS), including transoral laser microsurgery (TLM) and transoral robotic surgery (TORS), provides access to subsites in the head and neck that have traditionally been difficult to approach. Minor salivary gland tumors, while relatively uncommon, are frequently malignant and can occur at sites in the oropharynx accessible by transoral eHNS. Presented here is the largest review to date of patients with minor salivary gland tumors of the oropharynx managed with transoral eHNS as primary or salvage therapy.

**Methods:**

A retrospective chart review was performed, including data from 20 patients with minor salivary gland tumors of the oropharynx managed with transoral eHNS at 2 tertiary, academic medical centers. Details of tumor pathology, margin analysis, adjuvant therapy, and an assessment of oncologic outcome were included.

**Results:**

The base of tongue was the most common tumor site (75%). Adenoid cystic carcinoma (ACC) accounted for most cases (35%), and negative margins were obtained in most (95%) through an endoscopic-only approach. Overall, 50% of patients received post-operative radiation therapy. Postoperative complications were limited, with one patient (5%) returning to the OR for control of post-operative oropharyngeal bleeding. On average follow-up of 36 months, 90% of patients were alive with no evidence of recurrence.

**Conclusion:**

In this experience, transoral eHNS provided a safe and consistent surgical approach to management of minor salivary gland malignancies, with low complication rates and good locoregional control. Thus, transoral eHNS may play a valuable role in the multi-disciplinary management of these malignancies.

**Trial registration number:**

None/not applicable

**Electronic supplementary material:**

The online version of this article (doi:10.1186/s41199-017-0024-2) contains supplementary material, which is available to authorized users.

## Background

Transoral endoscopic head and neck surgery (eHNS) provides a minimally invasive approach for the surgical management of patients with tumors of the oropharynx [1].

Transoral laser microsurgery (TLM) is an invaluable component in the management of upper airway malignancies, and the clinical utility of transoral robotic surgery (TORS) is becoming increasingly well-established [1–7]. Both techniques provide means of accessing a range of anatomic sites in the head and neck that have traditionally been difficult to approach, and the growing familiarity with TORS has led to a rapid expansion of its indications [1, 4, 6, 8–14]. TORS is currently being used in the management of early-stage tumors, more advanced malignancies, tumor recurrences, deep neck space infections, tumors of unknown primary site, and comparatively benign conditions such as obstructive sleep apnea [1, 15–18]. The incidence of postoperative complications seen with TORS is generally low and has the potential to be further reduced by increased experience with the technique and an appreciation of risk factors for the most common issues [5, 19–22]. TLM has well documented acceptable oncologic outcomes, and interim functional and disease-control data have indicated that TORS can additionally be a safe, useful, and cost-effective component in the approach to a variety of conditions in the head and neck [5, 10, 17, 23–26]. There are ongoing efforts to further document the value of both TLM and TORS and expand the clinical application of these techniques.

Minor salivary gland tumors, while relatively uncommon, are frequently malignant [27–30]. They most often present at the hard palate, but can occur at a range of sites in the oral cavity, oropharynx, and sinonasal region that are difficult to access [27–32]. Complete surgical resection is recommended, often followed by adjuvant radiation, except in early stage tumors excised with negative margins with no adverse pathological features [30, 33–37]. Margin status has indeed been shown in several studies to be an independent predictor of postoperative survival, illustrating the importance of primary surgical intervention with definitive intent [35, 38–41]. Achieving negative margins can be especially challenging when certain sites, such as the oropharynx, are involved. Even traditional approaches can leave an appreciable percentage of patients with positive or close margins, and these techniques can often be associated with significant morbidity and the need for extended reconstructive efforts [32, 33, 35, 38, 40]. There is thus appreciable interest in developing novel therapeutic strategies for these malignancies [30].

This article represents the largest series, to date, of minor salivary gland tumors of the oropharynx managed with transoral eHNS – either TLM or TORS and serves to further demonstrate the utility of transoral techniques in the multi-disciplinary approach to these tumors.

## Methods

### Patient population

Patients who underwent evaluation and treatment for minor salivary gland tumors of the oropharynx at two independent academic medical centers (M.D. Anderson Cancer Center and Stanford University) from 2007 to 2013 were eligible for review. Patients included were those who underwent surgery and for whom complete data on demographics, pathology, treatment, and follow-up were available (Additional file 1: Table S1). Exclusions were made when available data was incomplete or the patient treatment course involved multiple, non-standard or extensive reconstructive procedures in addition to endoscopic surgery.

### Treatment

#### Transoral laser microsurgery (TLM) and transoral robotic surgery (TORS)

TLM/TORS was performed primarily by 4 surgeons (M.E.K, A.C.H, F.C.H. and E.J.D.) using either a free-beam or fiberoptic carbon dioxde (CO_2_) laser (TLM) or a robotic surgical system (Intuitive Surgical Inc) as previously described [1, 4, 19]. For TLM, a wide-mouthed laryngoscope was introduced transorally and the CO2 laser was then used to achieve oncologic resection with grossly negative margins, confirmed when appropriate with frozen-section pathology. In this series, the resection was performed using an en-bloc approach for TLM, rather than a piecemeal approach. For TORS, either the Feyh-Kastenbauer, Crowe-Davis, or Dingman retractors were used to provide access to the oropharynx and the robotic surgical system was brought into position and docked in a standard fashion. The 5-mm spatulated cautery tip was then used to achieve oncologic resection with gross negative margins, confirmed when appropriate with frozen-section pathology. Intraoperative tracheostomy or placement of a feeding tube was also performed at the discretion of the treating surgeon, with postoperative decannulation or feeding tube removal guided by clinical assessment (i.e. tolerance of cap trial, ability to tolerate adequate oral intake). The administration of adjuvant radiation or radiation was guided by standard institutional practice, with general indications for radiation being adverse pathologic features (i.e. perineural or lymphovascular invasion, positive or close margins) [30, 35, 42]. Radiation was 60–70 Gy given in standard fraction as previously reported [42].

### Data acquisition/statistical methods

Data was collected in the form of a standardized worksheet, de-identified of any personal information. Included in the worksheet were basic demographic information (i.e. age and sex), along with primary tumor site, primary tumor pathology, involvement of additional sub-sites, clinical and pathological tumor staging, treatment, the use of a tracheostomy and/or feeding tube, assessment of outcome at most recent clinic visit, and documentation of postoperative complications. Length of follow-up was calculated from date of surgery to most recent clinic visit.

## Results

### Patient demographics and tumor characteristics

**Fig. 1:**
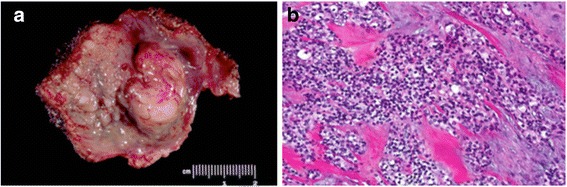
Hyalinizing clear cell carcinoma. **a** Submucosal, rubbery, firm tumor of base of tongue. **b** Morphology (hematoxilin and eosin) reveals nests, trabeculae, and islands of clear cells embedded in a hyalinized, fibrotic stroma (Figures courtesy of Diana Bell, MD)

**Fig. 2:**
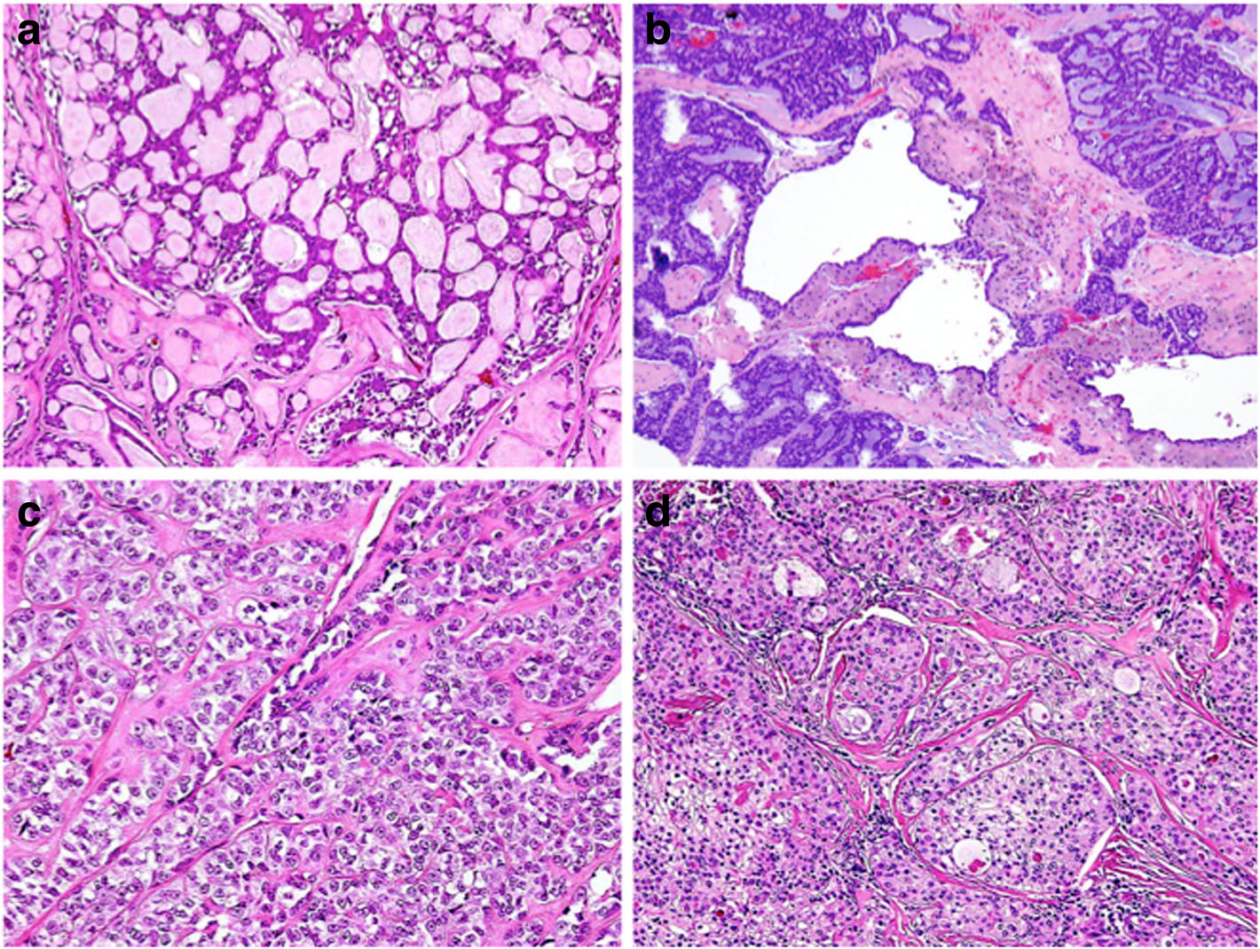
Representative histological sections (hematoxilin and eosin stain) of minor salivary gland tumors found in the oropharynx in this series. **a** Adenoid cystic carcinoma (predominantly cribriform pattern). **b** Polymorphous-type adenocarcinoma. **c** Myoepithelial carcinoma, clear cell variant. **d** Mucoepidermoid carcinoma, intermediate grade (Figures courtesy of Diana Bell, MD)

Twenty patients were included in the study. The average age was 61 and there were slightly more women than men (Table 1). The most common tumor site was the base of tongue (75%), but half of the patients included in the analysis had involvement of additional sub-sites (Table 1), such as the floor of mouth, glossopharyngeal sulcus, and lateral pharyngeal wall. The majority of patients (80%) were clinically staged as either T1 or T2 (Table 1). One patient who initially underwent an incisional biopsy of a mucoepidermoid carcinoma involving the base of tongue had no identifiable lesion on assessment at referral, was clinically staged as Tx, eventually developed a clinically visible lesion on follow-up, and underwent a limited base of tongue resection through TORS. This patient was was staged on final pathology as T1, did not receive postoperative radiation, and was alive with no evidence of disease at 91.8 months of follow-up. Two patients with clinical T4 adenoid cystic carcinomas of the base of tongue were downgraded on final pathology to either T2 or T3. Adenoid cystic carcinoma was the most common histologic subtype, though there was appreciable diversity in tumor pathology within this series (Table 1, Figs. 1 and 2).

**Table 1:** Patient demographics and tumor characteristics

Average Age (Range)	61 (43 to 84)
Sex	8 Males; 12 Females
Primary Tumor Site	
Base of Tongue	15 (75%)
Soft Palate	2 (10%)
Tonsil/Tonsillar fossa	3 (15%)
Involvement of additional/adjacent sites	10 (50%)
Clinical Staging	
Tx	2 (10%)
T1	8 (40%)
T2	8 (40%)
T4	2 (10%)
Pathology	
Adenoid Cystic Carcinoma	7 (35%)
Clear Cell Carcinoma	5 (25%)
Mucoepidermoid Carcinoma (intermediate grade)	4 (20%)
Polymorphous low-grade adenocarcinoma	3 (15%)
Myoepithelial Carcinoma	1 (5%)

### Treatment and additional perioperative management

Of the 20 patients included in the analysis, 10 underwent TORS along with postoperative radiation (Table 2). Frontline transoral endoscopic head and neck surgery (TORS or TLM) was used as the only treatment for 9 patients. One patient with a T2 clear cell carcinoma of the base of tongue underwent primary radiation, recurred, and subsequently underwent salvage resection with TORS. This patient was alive with no evidence of disease at 25 months of follow-up. Only one patient with a myoepithelial carcinoma of the base of tongue with a cN0 neck underwent a bilateral levels 2–4 neck dissection at the discretion of the treating surgeon.

**Table 2:** Treatment and additional perioperative management

Primary treatment	
TORS, with post-operative radiation therapy	10 (50%)
TORS alone	7 (35%)
TLM alone	2 (10%)
Salvage TORS after radiation therapy	1 (5%)
Negative margins	19 (95%)
Perioperative Feeding Tube, temporary	13 (65%)
Perioperative Feeding Tube, permanent	0 (0%)
Perioperative Tracheostomy	4 (20%)

Of the 9 patients treated with transoral eHNS alone, there were 4 with mucoepidermoid carcinomas (all intermediate grade), 3 with clear cell carcinomas, and one each with either a myoepithelial carcinoma or polymorphous low-grade adenocarcinoma. There were no patients with adenoid cystic carcinomas who were managed with surgery alone. All patients treated with surgery alone had negative margins on final pathology and were alive with no evidence of disease at most recent follow-up.

Most patients with adenoid cystic carcinomas (86%) had negative margins on final pathology, and all received postoperative radiation. One of these patients with a clinical T4, pathological T2 adenoid cystic carcinoma involving the base of tongue and floor of mouth was resected by TORS, found to have positive margins on final pathology, underwent postoperative radiation therapy, and was alive with no evidence of disease at 12.8 months of follow-up. Another patient with a T2 adenoid cystic carcinoma of the tonsil and soft palate developed distant disease in the lungs after surgery with negative margins received postoperative radiation therapy; this patient was alive with known pulmonary metastatic disease at 24 months of follow-up.

A total of 13 patients required a temporary perioperative feeding tube. There was no clear association between the need for a feeding tube and radiation – 8 of the patients who underwent radiation required a feeding tube as well as 5 of the patients did not receive radiation. Four patients received tracheostomy: both patients with T4 tumors, a patient undergoing salvage TORS after radiation. A single patient who underwent tracheostomy without receiving radiation therapy had a clinically T2 clear cell carcinoma of the base of tongue/glossopharyngeal sulcus with microscopic extension into the neck with exposure of the course of the lingual artery and external carotid. Due to the anticipated risk of bleeding, decision was made to perform a tracheostomy during the initial operation. The patient experienced a self-limited episode postoperative hemorrhage on postoperative day 6 necessitating subsequent embolization without surgical intervention. This patient was eventually decannulated 3 weeks later and was alive with no evidence of disease at 22.7 months of follow-up.

### Patient outcomes

Average follow-up was 36 months (range 5.1 to 100.4 months), and 90% of patients were alive with no evidence of disease (Table 3). As mentioned, one patient with a T2 adenoid cystic carcinoma of the tonsil and soft palate had negative margins on initial resection and received postoperative radiation therapy, and was alive with pulmonary metastases at 24 months of followup. Another patient with a T2 adenoid cystic carcinoma of the base of tongue underwent TORS and then postoperative radiation and had no evidence of disease at 5.1 months of follow-up, but subsequently died of metastatic ovarian adenocarcinoma.

**Table 3:** Patient Outcomes

Average months of follow-up (range)	36 (5.1 to 100.4)
Patient Status	
Alive, no evidence of disease	18 (90%)
Alive, distant metastasis	1 (5%)
Deceased, other causes	1 (5%)
Complications	2 (10%)
Oropharyngeal bleeding	1 (5%)
Readmission for dehydration	1 (5%)

Only one patient developed clinically significant oropharyngeal bleeding. A patient with clinical T4 adenoid cystic carcinoma of the base of tongue and floor of mouth underwent TORS and had a bleed on postoperative day 10, necessitating operative control. This patient subsequently decannulated and underwent postoperative radiation, tolerating an oral diet at 12.8 months of follow-up. One patient with a T1 adenoid cystic carcinoma of the base of tongue underwent TORS, had an intraoperative feeding tube placed that was removed on postoperative day 1 prior to discharge, but was readmitted on postoperative day 2 with dehydration. At the time of last follow-up, the patient was tolerating an oral diet and alive with no evidence of disease.

## Discussion

This study further demonstrates the safety of transoral eHNS (both TLM and TORS) in approaching tumors of the oropharynx and adds to current data demonstrating the efficacy of these techniques in the management of minor salivary gland malignancies [43]. The data presented here compare favorably to another series that described the use of TORS to manage patients with either T1 or T2 minor salivary gland tumors of the oropharynx [43]. Mucoepidermoid carcinoma was most frequently encountered in this experience and only 30% of patients were found to have close margins (<5 mm). Radiation therapy was given to 40% of patients and most did well: at an average follow-up of 24 months, 70% of patients were disease-free [43]. Our series adds to this data, presents a more diverse set of pathology, and extends follow-up and further demonstrates the value of transoral eHNS in minor salivary gland tumors of the oropharynx.

While appreciably less common than oropharyngeal squamous cell carcinoma, minor salivary gland tumors are not infrequently found in the oropharynx. The majority are malignant and surgical resection with curative intent is recommended as the frontline therapy [30]. While most tumors in this study were adenoid cystic carcinoma, there was considerable diversity in pathology (Table 1, Figs. 1 and 2). Clear cell carcinomas, mucoepidermoid carcinomas, polymorphous low-grade adenocarcinomas, and myoepithelial carcinomas were found in a variety of sites in the oropharynx. The base of tongue was the most common primary site, though half of patients were found to have involvement of additional subsites.

Several studies have reiterated the importance of complete surgical resection through documenting poor outcomes when margins return as positive [40, 41]. The majority of patients (95%) were found to have negative margins on final pathology. However, in one case in this series, a surgeon encountered extensive submucosal spread along presumed lymphangitic and/or perineural channels. Despite several attempts at re-exicision, the surgeon elected to defer further resection and potential morbidity given the need post-operatively for radiation therapy. At more than one year of follow-up after all treatent, the patient was alive, tolerating a regular diet with no evidence of disease at most recent.

Adjuvant radiation therapy to the primary site is recommended for most patients, but in very select cases might be omitted. Frontline surgery might be considered in patients with early-stage disease resected with clear margins and no adverse features on pathology [30]. Many patients in this series received adjuvant radiation therapy, with others declining radiation therapy (1), undergoing neoadjuvant radiation (1), or having T1/T2 lesions that were completely excised with negative margins and had favorable pathology without adverse features.

In addition to providing acceptable oncologic outcomes, transoral eHNS has the potential to avoid morbidity typically associated with more traditional approaches [1]. While most patients in this series had a temporary feeding tube, few had prolonged dependence, generally less than 2–3 weeks. These data compares well with a recent report, as well as other data, documenting functional outcomes following TORS, with a similar number of patients requiring prolonged enteral access and most reporting a favorable MDADI score [26, 43–45]. Additionally, the need for a tracheostomy was limited, and all patients who underwent tracheostomy at the time of initial resection were decannulated within 1 month of surgery.

The cumulative incidence of complications following TORS varies with surgical experience, but is generally near 10%, with postoperative hemorrhage accounting for approximately 3% of these adverse events [20]. Other studies have documented postoperative bleeding in up to 7.5% of patients following TORS for a variety of indications [21]. Other complications were relatively uncommon, with only 1 patient needing to be readmitted for postoperative dehydration.

In our series, a single patient was taken to the operating room for management of hemorrhage. Of note, bleeding occurred at a median of 8 days after the initial operation in this report, and was significantly associated with the postoperative use of antithrombotic medications [21]. A thorough preoperative assessment of the need for antithrombotic therapy may limit this complication and further increase the safety profile of TORS. Bleeding complications have also been documented with TLM, with 1.4% of patients experiencing clinically significant bleeding in one recent TLM series [46].

For most patients with squamous cell carcinoma, a neck dissection is indicated for management of the cervical lymphatics, during which ligation of the at-risk arterial supply of the tumor is a critical aspect of the surgery. Several series now have shown that ligation of branches of the external carotid artery (ECA) may reduce the incidence of severe adverse events of bleeding [46, 47]. However, depending on histology, such as adenoid cystic carcinoma, oropharyngeal tumors of salivary gland origin may not require cervical lymphadenectomy. Based on recent data, we would still recommend neck dissection for patients with OPC of salivary gland origin in order to undergo ligation of these branches of the ECA or elective arterial embolization to reduce the potential risk of oropharyngeal hemorrhage.

Two patients had outside complete excisional biopsies for their salivary gland tumors with no apparent disease left behind. Both of these patients required re-resection after a long period of observation, surveillance, and serial imaging. Both patients had their disease under control but the absence of obvious tumor after the initial biopsy delayed definitive treatment. One potential observation from this experience is that surgeons who first diagnose these tumors might consider tumor mapping or incisional biopsy of these lesions, rather than an excisional biopsy. Complete removal for diagnostic purposes may create delay and difficulty for the patient and surgeon. With new surgical techniques in transoral eHNS, patients with gross disease remaining after diagnostic biopsy can proceed to definitive treatment with a shorter observation period.

## Conclusions

This series adds to a growing body of literature documenting the utility of transoral endoscopic head and neck surgery in managing not only oropharyngeal SCC, but other malignant and benign conditions of the upper aerodigestive tract [1]. Transoral endoscopic head and neck surgery can be a valuable component in the multidisciplinary management of minor salivary gland tumors of the oropharynx and its use should be considered in an initial approach to resection.

## Additional file

Additional file 1: Table S1. Original data on patients included in report. (XLSX 35 kb)

## Abbreviations

ACC: Adenoid cystic carcinoma
ECA: External carotid artery
eHNS: Endoscopic head and neck surgery
TLM: Transoral laser microsurgery
TORS: Transoral robotic surgery
